# Processing Metrical Information in Silent Reading: An ERP Study

**DOI:** 10.3389/fpsyg.2016.01432

**Published:** 2016-09-22

**Authors:** Olga Kriukova, Nivedita Mani

**Affiliations:** Psychology of Language Research Group, Department of Psychology, Georg-Elias-Müller Institute of Psychology, Georg-August-Universität GöttingenGöttingen, Germany

**Keywords:** speech meter, metrical congruence, ERPs, N400, N325

## Abstract

Listeners are sensitive to the metric structure of words, i.e., an alternating pattern of stressed and unstressed syllables, in auditory speech processing: Event-related potentials recorded as participants listen to a sequence of words with a consistent metrical pattern, e.g., a series of trochaic words, suggest that participants register words metrically incongruent with the preceding sequence. Here we examine whether the processing of individual words in silent reading is similarly impacted by rhythmic properties of the surrounding context. We recorded participants’ EEG as they read lists of either three trochaic or iambic disyllabic words followed by a target word that was either congruent or incongruent with the preceding metric pattern. Event-Related Potentials (ERPs) to targets were modulated by an interaction between metrical structure (iambic vs. trochaic) and congruence: for iambs, more positive ERPs were observed in the incongruent than congruent condition 250–400 ms and 400–600 ms post-stimulus, whereas no reliable impact of congruence was found for trochees. We suggest that when iambs are in an incongruent context, i.e., preceded by trochees, the context contains the metrical structure that is more typical in participants’ native language which facilitates processing relative to when they are presented in a congruent context, containing the less typical, i.e., iambic, metrical structure. The results provide evidence that comprehenders are sensitive to the prosodic properties of the context even in silent reading, such that this sensitivity impacts lexico-semantic processing of individual words.

## Introduction

Metrical stress, patterns of alternations of strong and weak syllables in polysyllabic words, phrases, or sentences, is an important characteristic of spoken language. Metrical regularities in speech have been shown to play an important role in language acquisition and segmentation of words from continuous speech for both infants (Jusczyk et al., 1993; Thiessen and Saffran, 2007) and adults (Sanders et al., 2002). Regularity in metrical structures has also been shown to facilitate phonological (Pitt and Samuel, 1990), lexico-semantic (Rothermich et al., 2012) and syntactic processing (Roncaglia-Denissen et al., 2013).

Given the functional importance of metrical stress for speech processing, it is not surprising that listeners register metrical regularities and inconsistencies even without explicit instructions to do so. Recent studies by Bohn et al. (2013) and Henrich et al. (2014) demonstrated that listeners prefer well-formed rhythmical structures, i.e., those consisting of alternating strong and weak syllables, and register deviation from this pattern when encountering two strong or two weak syllables in a row. Another study by Böcker et al. (1999) presented participants with sequences of four disyllabic Dutch words where the last word either followed the same metrical pattern as the preceding words or deviated from it. They observed an event-related potential (ERP) component they termed the N325, which was sensitive to both metrical congruence and structure processing. The negativity was enhanced for critical items incongruent with the preceding metrical structure. The effect was observed in a passive listening task, but increased when participants were explicitly asked to attend to the metrical properties of the presented stimuli. In other words, the process of registering metrical properties of speech is, as suggested by the authors, both endogenous and exogenous in nature. Moreover, the N325 was larger for iambic words, consisting of a weak syllable followed by a strong one, compared to trochaic words, which follow a strong-weak pattern. Böcker et al. (1999). proposed that this reflects listeners’ sensitivity to the typical metrical pattern of their native language (in Dutch, the trochee is the predominant metrical pattern). Importantly, its modulation by metrical pattern distinguished the behavior of the N325 from the N400, an ERP index of lexico-semantic processing, that was sensitive to only metrical incongruences. Taken together, the findings of Böcker et al. (1999). indicated that there may be an independent cognitive process of registering metrical properties of speech (both rhythmic incongruities and metrical typicality) that at later stages interferes with lexico-semantic processing.

While some studies have argued that a process indexed by the N325 is an instance of lexico-semantic processing (Knaus et al., 2007; Magne et al., 2007), recently, Rothermich et al. (2010) reported evidence for a neural process related to metrical processing. Participants listened to jabberwocky sentences that were devoid of any semantic content but preserved the morphological and syntactic properties of real language. The sentences followed a consistent metrical pattern and implicitly prompted participants to generate rule-based predictions about the stress-patterns of the upcoming words. Targets that violated the metrical structure of the preceding context elicited an enhanced negativity around 200–350 ms. Given that the negativity occurred in the absence of any lexico-semantic content, Rothermich et al. (2010) suggested that it reflects a domain-general process of registering structural violation of rule-based sequences.

The present study investigated whether participants are similarly sensitive to metrical structure during silent reading. A growing body of recent research has demonstrated that visual language processing covertly activates phonological representations at different levels: individual phonemes (Frost, 1998), sub-phonemic properties such as phonetic length (Abramson and Goldinger, 1997) and supra-segmental features such as prosodic phrase boundaries (Steinhauer and Friederici, 2001; Roll et al., 2012; Schremm et al., 2015). Word stress is likewise activated in silent reading (Ashby and Clifton, 2005), which manifests as interference with the orthographic and lexico-semantic processing of these words (Harris and Perfetti, 2016; Kriukova and Mani, 2016). For instance, Kriukova and Mani (2016) observed an increased N400 and P600 in response to orthographic misspellings embedded in stressed syllables compared to misspellings in unstressed syllables when these occurred in the middle of a word. We suggested that even in silent reading misspellings in strong syllables are more salient and disrupt lexical access more than misspellings in weak syllables, which in turn leads to active re-evaluation reflected in the enhanced P600.

While these studies examine the processing of phonological stress in individual words, Breen and Clifton (2011, 2013) investigated metrical processing of words in the context of larger structures, i.e., sentences, across a series of eye-tracking experiments. In one experiment, participants read sentences that contained words ambiguous with regard to the part of speech (noun vs. verb). They observed that syntactic reanalysis took longer when concurrent metrical reanalysis had to be performed, i.e., changing a word form from noun *ABstract* to a verb *abSTRACT* in comparison when no metrical reanalysis had to be done (e.g., noun *rePORT* vs. verb *rePORT*). A similar observation for metrical reanalysis costs was made when words were embedded into limericks suggesting that metrical information is monitored and interacts with other forms of processing in silent reading.

Yet, it remains unclear whether perception of metrical patterns in silent reading constitutes a separate process, or whether it is only reflected in interaction with other, e.g., lexico-semantic processes. The latter possibility is particularly plausible given that visual word processing does not require overt speech and is not accompanied by explicit acoustic processing. Against this background, the present experiment examined metrical processing in silent reading using the design of Böcker et al. (1999), with the distinction that participants were tasked with silently reading nouns as they performed semantic judgments on occasional words to maintain their attention. In particular, we asked whether readers register metrical incongruences during silent reading and whether these may impact lexical processing of metrically congruent vs. incongruent words. In addition, we have explored whether participants show differential processing of metrical patterns by comparing sensitivity to iambic and trochaic metrical structures.

Participants (*n* = 19) in the present study were instructed to silently read sequences of words that they saw on a screen. Every word sequence included four words where the fourth target word was metrically congruent or incongruent with the preceding words. ERPs were time-locked to the onset of the target words. In the absence of overt speech and behavioral responses, ERPs provide a perfect tool for registering differences in the neural processing of different types of stimuli. If participants were sensitive to metrical differences during silent reading as in acoustic processing as in Böcker et al. (1999), we expected ERPs to be modulated by metrical information in the time window from about 200–400 ms. Furthermore, were metrical information to impact lexico-semantic processes, modulation of the N400, from about 400–600 ms, should also be observed.

## Materials and Methods

### Participants

Twenty-four native German speakers were recruited from the student population of the University of Göttingen to take part in the experiment for course credits or financial reimbursement. The experiment was approved by the Department Ethics committee and written informed consent was provided by every participant prior to the experiment. The data from one participant were discarded from the analyses due to technical problems during the recording and the data from further three participants were eliminated due to a low number of artifact-free trials (less than 20 trials per condition). After examining performance on comprehension questions, the data from another participant were eliminated as he/she provided correct answers only on 37% of comprehension questions making it unclear whether this participant attended to the presented words.

The remaining 19 participants (11 male) were on average 24 years old (aged 18–30), right-handed and had normal or corrected-to-normal vision.

### Materials

A total of 800 common German nouns were selected from CELEX database. Of these, 160 disyllabic words beginning with a consonant were employed as critical targets. The selected words followed a clear stress pattern with half of these words being trochees (i.e., stressed on the first syllable) and the other half being iambs (stressed on the second syllable) not allowing for alternative stress placement. Trochaic and iambic words differed neither in their mean frequency of occurrence per million (trochaic: *M* = 16, range: 0–85); iambic: *M* = 17, range: 0–77), *t*(158) = 0.186, *p* = 0.853, nor mean word length (trochaic: *M* = 6, range: 4–8 letters; iambic, *M* = 6, range: 5–10), *t*(158) = 1.453, *p* = 0.148. Two hundred and forty iambic and 240 trochaic disyllabic words were selected to create metric contexts for the targets, and 160 monosyllabic nouns functioned as distractors.

Every trial started with three metric primes, which were either all trochaic or iambic followed by a target that was either metrically congruent or incongruent with the preceding three word primes. This resulted in four target conditions as exemplified in **Table 1**: trochaic congruent, trochaic incongruent, iambic congruent, iambic incongruent. Every experimental sequence was followed by a filler word. In 90 trials, fillers were followed by a comprehension question where participants were asked to decide whether the preceding word could be fitted into a shoebox or not. Forty five questions required a positive and 45 a negative answer. These questions emphasized semantic processing and were introduced (i) to ensure that participants read for understanding and (ii) to distract participants’ attention from the critical manipulation.

**Table 1:** Example of stimuli used in four experimental conditions.

Prime 1	Prime 2	Prime 3	Target	Target Condition
H**a**mster *“hamster”*	Br**i**lle *“glasses”*	Sp**ü**hlung *“conditioner”*	B**a**gger *“digger”*	Trochaic congruent
Kult**u**r *“culture”*	Geh**i**rn *“brain”*	Pal**a**st *“palace”*	B**a**gger *“digger”*	Trochaic incongruent
Kult**u**r *“culture”*	Geh**i**rn *“brain”*	Pal**a**st *“palace”*	Balk**o**n *“balcony”*	Iambic congruent
H**a**mster *“hamster”*	Br**i**lle *“glasses”*	Sp**ü**hlung *“conditioner”*	Balk**o**n *“balcony”*	Iambic incongruent

*vowels in stressed syllables are highlighted in bold.*

Target words were divided into 4 item lists. Assignment of item lists to four experimental conditions was counterbalanced across participants. Thus any difference in ERPs across congruent and incongruent conditions can only be attributed to the difference in the context preceding the presented words and not to the individual words in each condition. Every participant received an individual stimulus list with a pseudo-randomized sequence of targets, primes, and fillers.

### Procedure

The experiment was programmed using Presentation Software. All words were presented centrally in black (Arial; size 54) on a light gray background on a 42” TV screen. Participants were seated at a viewing distance of 100 cm from the screen. They were asked to read the words silently paying attention to every word in order to correctly respond to occasional semantic questions. Responses to questions were provided by pressing one of the buttons on an X-Box controller.

Every trial sequence (consisting of three prime words, a target word, a filler word, and sometimes a comprehension question) started with a fixation cross that remained on the screen for 1500 ms. Next, three primes, a target trial and a filler trial were displayed sequentially for 600 ms each with an inter-stimulus interval of 800 ms. In 90 trial sequences, untimed comprehension questions followed the filler trial requiring participants to press yes- or no- buttons on the X-Box controller. Participants worked through a total of 160 trial sequences divided into 5 blocks separated by self-paced breaks.

### Electrophysiological Recordings and Analyses

BioSemi Active Two was used to record continuous EEG from 32 silver/silver-chloride electrodes placed into the elastic cap according to the extended International 10–20 system. Furthermore, EEG from two other electrodes placed at each mastoid was recorded to allow off-line re-referencing to the averaged mastoid signals. Oculomotor activity was estimated using signals from EOG (electrooculography) with two electrodes placed on the outer canthi of both eyes and one electrode below the left eye. Data were acquired with an amplifier bandpass from DC to 400 Hz and digitized at a sampling rate of 2048 Hz with a resolution of 24-bit. Electrode offsets were kept below 25 μV. Oﬄine processing of the data was conducted with BESA. This included filtering the data re-referenced to averaged mastoids with a band-pass filter (0.01–30 Hz), resampling the data to 250 Hz, splitting the signal into individual epochs from 200 ms pre-stimulus to 800 ms post-stimulus, and correcting the waveforms relative to the 200 ms pre-stimulus baseline period. Artifact-containing trials were rejected if the peak amplitude in an epoch exceeded a cut-off threshold determined individually for each participant following automatic artifact-screening procedure (minimal threshold was +/-70 μV in any of the channels). A minimum of 32 artifact-free trials contributed to individual subject grand averages in each of the four conditions (trochaic congruent and incongruent, iambic congruent, and incongruent). A 20-Hz low-pass filter was applied to ERP waveforms for the purpose of illustration only.

**FIGURE 1:**
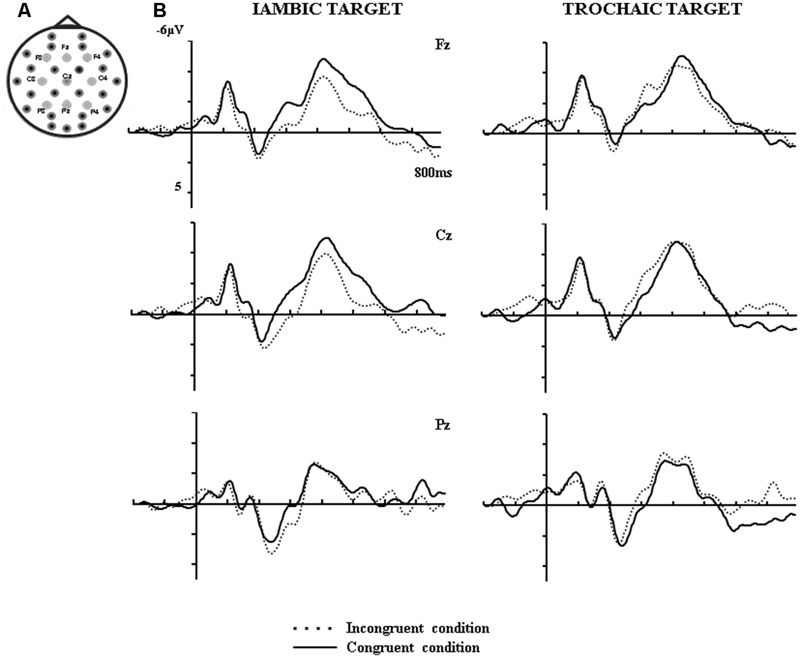
**(A)** Regions of interest selected for the ERP analysis. **(B)** Grand average ERPs at three representative electrodes, Fz, Cz and Pz, elicited by trochaic/iambic targets that were either congruent or incongruent with preceding metrical context.

To capture the distribution of the metrical and lexico-semantic effects in reading as reported in previous studies (Böcker et al., 1999; Kriukova and Mani, 2016), the following 9 representative electrodes were selected for the analysis: F3, Fz, F4, C3, C4, Cz, P3, Pz, and P4 as depicted in **Figure 1A**. Repeated measures analyses of variance (ANOVAs) were employed for the inferential statistics. *F*-values corrected for non-sphericity with Huyn-Feldt procedure are reported together with uncorrected degrees of freedom and corrected p-values. Probability values of the subsequent t-tests are reported after correcting for multiple comparisons using Holm–Bonferroni procedure. Partial eta squared and Cohen’s d are reported as indicators of effect sizes for ANOVAs and *t*-tests, respectively.

## Results

To ensure that participants read words presented during the experiment for comprehension, accuracy of answers to comprehension questions was analyzed. The mean accuracy was 75% (ranging from 55 to 81%).

Visual inspection of the pattern of the data (**Figure 1B**) suggests that metrically congruent words evoked more negative ERPs than metrically incongruent ones in the 250–400 and 400–600 ms time windows. The difference between congruent and incongruent conditions appears larger in the iambic than trochaic condition. Furthermore, these effects extend beyond 600 ms and are also visible in the 600–800 ms time window. To evaluate the results statistically, separate four-way ANOVAs with within-subject factors Meter (trochee; iamb), Congruence (metrically congruent with preceding primes; metrically incongruent), Location (frontal electrode line, central, parietal) and Laterality of the electrode (left, midline, right) were conducted on the mean voltages evoked by targets in the 250–400 ms (N325), 400–600 ms (N400), and 600–799 ms time windows. Only significant effects and interactions involving factors Meter and Congruence are reported.

In the early time window, there was a four-way interaction, *F*(4,72) = 3.695, *p* = 0.009, $\eta_{p}^{2}$ = 0.17. We then conducted two ANOVAs with factors Congruence, Location, and Laterality on the data from iambic and trochaic conditions separately. The ANOVA on the data from the iambic condition yielded a marginally significant effect of Congruence, *F*(1,18) = 3.936, *p* = 0.063, $\eta_{p}^{2}$ = 0.179, indicating a tendency of congruent items to evoke more negative potentials. No effect of Congruence or interactions involving this factor were found in the trochaic condition.

In the N400 time window, the main ANOVA revealed a marginally significant effect of Congruence, *F*(1,18) = 3.387, *p* = 0.082, $\eta_{p}^{2}$ = 0.158, and interaction between Congruence and Location, *F*(2,36) = 3.685, *p* = 0.052, $\eta_{p}^{2}$ = 0.17, suggesting a tendency toward more negative ERPs to targets in the congruent condition, especially over central electrode line, *t*(18) = 2.311, *p* = 0.033, *d* = 0.526. Importantly, the results also yield a four-way interaction, *F*(4,72) = 3.378, *p* = 0.015, $\eta_{p}^{2}$ = 0.158. Subsequently, trochaic and iambic conditions were tested separately. In the iambic condition, the three-way ANOVA revealed a main effect of Congruence, *F*(1,18) = 6,862, *p* = 0.017, $\eta_{p}^{2}$ = 0.276, and an interaction between Congruence and Location, *F*(2,36) = 4.099, *p* = 0.039, $\eta_{p}^{2}$ = 0.185. Individual comparisons revealed that the negativity for incongruent items was significantly smaller than for congruent items over frontal and central electrode lines, *t*(18) = 3.250, *p* = 0.004, *d* = 0.746, and *t*(18) = 2.684, *p* = 0.03, *d* = 0.616, respectively. Three-way ANOVA on the data from the trochaic condition revealed a marginal interaction between Congruence, Location and Laterality, *F*(4,72) = 2.379, *p* = 0.065, $\eta_{p}^{2}$ = 0.116. Subsequent two-way ANOVAs with factors Congruence and Laterality conducted separately for the data from the frontal, central, and parietal locations, however, failed to uncover any effects or interactions with Congruence.

In the late time window, 600–799 ms, there was likewise a four-way interaction between Meter, Congruence, Location and Laterality, *F*(4,72) = 3.695, *p* = 0.009, $\eta_{p}^{2}$ = 0.17. The analysis conducted on the data from the iambic condition revealed a marginally significant effect of Congruence, *F*(1,18) = 4,053, *p* = 0.059, $\eta_{p}^{2}$ = 0.184, and a three-way interaction, *F*(4,72) = 2.867, *p* = 0.049, $\eta_{p}^{2}$ = 0.137. To resolve it, two-way ANOVAs with factors Congruence and Laterality were conducted on the data from frontal, central, and parietal electrode lines. These revealed an interaction of Congruence and Laterality, for the central electrode line, *F*(2,36) = 5,03, *p* = 0.012, $\eta_{p}^{2}$ = 0.218, as well as marginally significant effect of Congruence for central and frontal locations, *F*(1,18) = 4,182, *p* = 0.056, $\eta_{p}^{2}$ = 0.189, and *F*(1,18) = 3,537, *p* = 0.076, $\eta_{p}^{2}$ = 0.164, respectively. For the central electrode line, enhanced positivity for incongruent items was especially pronounced over Cz, *t*(18) = 2.771, *p* = 0.013, *d* = 0.104. No effect of Congruence or interactions with this factor were observed in the trochaic condition.

## Discussion

The goal of the present experiment was to investigate whether readers are sensitive to metrical patterns of the spoken language – such as metrical incongruities and metrical structure – during silent reading and whether metrical processing interacts with lexico-semantic visual word processing. The analyses focused on the two components previously observed in the literature: the N325, a component known to be sensitive to metrical properties in spoken language processing (Böcker et al., 1999; also Rothermich et al., 2010) and the N400, a component related to lexico-semantic processing but shown to vary as a function of metrical characteristics of speech (Böcker et al., 1999; Knaus et al., 2007). Both metrical variables, i.e., metrical structure and metrical congruence, modulated ERPs in the N325 time window of 250–400 ms, showing a tendency for attenuated negativity in the incongruent compared to congruent items. This effect was clearly pronounced in the N400 time window from 400 to 600 ms. The N400 was in general modulated by metrical Congruence, which primarily was driven by statistically reliable differences between iambic congruent and incongruent items with a fronto-central distribution of the effect. These effects extended beyond 600 ms and were also observed in the later time window from 600 to 799 ms.

The critical time windows set *a priori* for the analyses in the present research have been chosen in accord with previous studies showing an early meter-related negativity to be different from the later emerging N400 (Böcker et al., 1999). The results demonstrate rather similar pattern of ERPs in both time windows, a finding which raises the question as to whether there are two different processes related to metrical processing or whether they both reflect the same underlying process. Lexico-semantic effects indexed by the N400 have been previously observed as early as 200 ms post target-onset and extending to 600 ms (for a review see Kutas and Federmeier, 2011) whereas a relatively short-lasting metrical negativity in speech processing (Böcker et al., 1999; Rothermich et al., 2010) was observed in an overlapping time window from about 200/240 ms to 350 ms. This leaves open the possibility that no pure meter-related process was observed in this experiment but the components modulated reflect a long-lasting complex of lexico-semantic processes modulated by the metrical properties of word lists. Previous studies suggested that even though there are distinct underlying brain networks for metrical and lexico-semantic processing (Rothermich and Kotz, 2013) and independent ERP manifestations of these under task instructions that direct participants’ attention toward semantic or rhythmic processing (Rothermich et al., 2012), both processes also interact with effects of semantic processing tasks overruling the ERP effects of pure metrical processes in auditory sentence processing (Magne et al., 2007; Astesano et al., 2004).

One further finding observed here is that ERP modulations similar to those in the N400 time window, were also pronounced from 600 to 799 ms. Several studies from auditory domain (Böcker et al., 1999; Marie et al., 2011; Rothermich et al., 2012) previously reported that modulations of early components by metrical properties are frequently accompanied by variations in the centro-parietally distributed Late Positive Component (LPC) interpreted as reflecting some kind of re-evaluative processes (Böcker et al., 1999; Marie et al., 2011; Rothermich et al., 2012). In principle, it is possible that participants implicitly registered metrical incongruence in the iambic condition which further led to some structural re-evaluation in this condition reflected in the enhanced positivity of incongruent iambs. We consider this explanation somewhat unlikely given that LPC has been previously observed in tasks where participants’ attention was explicitly directed toward metrical evaluation of auditory input but not in tasks where metrical information was not capitalized. Moreover, the remarkable similarity of the late effect observed here to the effect in the N400 time window, including similar fronto-central distribution, indicates that it may reflect long-lasting N400 effects rather than a separate component. Indeed, in what follows, we similarly interpret the early N325-like effect as well as the N400.

The results of the present study add to a body of previous findings suggesting activation of various types of phonological information in silent reading (Abramson and Goldinger, 1997; Frost, 1998; Steinhauer and Friederici, 2001; Ashby and Clifton, 2005; Breen and Clifton, 2011; Kentner and Vasishth, 2016) and demonstrate readers’ sensitivity to the metrical properties of written language. The pattern of modulation of the ERPs seems, however, at odds with what has been previously observed in auditory speech processing. Metrically incongruent conditions have been shown to enhance negativity in the N325 time window (Böcker et al., 1999; Magne et al., 2007; Rothermich et al., 2010; Bohn et al., 2013; Henrich et al., 2015) and beyond that (Magne et al., 2016), which has been attributed to detection of metrical inconsistencies or violations of metrical structure. Deviations in the metrical structure, for instance, explicit violations such as lengthening of the syllable (Magne et al., 2007) or incorrect stress placement (Knaus et al., 2007; Bohn et al., 2013; Henrich et al., 2015), have also been shown to enhance the N400 relative to correct conditions indicating increased costs in lexical retrieval perhaps due to the perception of these words as pseudowords.

In contrast, in the current study, the negativity was enhanced when targets appeared in the congruent condition relative to the incongruent condition. Next, we examine potential reasons for this apparent difference between the current study and the results of previous studies on this issue to-date. We note that no auditory stimuli were presented to participants in the current experiment rendering the phonological manipulations subtle and unnoticed by participants (according to their reports). This is in contrast to previous studies which presented participants with auditory stimuli and also explicitly directed their attention to phonological processing. This explicit direction of attention has also been shown to increase the effects of metrical deviations relative when no explicit instructions are given. We also note that visual inspection of the results of Böcker et al. (1999) suggests that – in the passive listening task – metrically incongruent items may have attenuated the N325 in contrast to metrically congruent ones – the pattern consistent with the current results.

How may we explain our finding that, in silent reading, metrical incongruences attenuate the negativity, and that this is especially the case when iambic targets follow a sequence of trochees? One possible explanation for this finding stems from the frequency of the different metrical structures in the participants’ native languages. In the case when iambs are preceded by a trochaic context, the context contains the metrical structure that is more typical in German (73% of German disyllabic words are stressed on the first syllable (Féry, 1998)). Furthermore, we note that processing may be facilitated for words following a more typical metrical pattern as suggested by the attenuation of the N325 for trochees compared to iambs in Dutch (Böcker et al., 1999). This would suggest that, in the incongruent iambic condition, the context primes ought to be processed more easily, which may make the more atypical iamb target more salient relative to when the iamb is presented against the context of the atypical iambs. Notably, we did not observe similarly statistically reliable effects of congruence for trochaic targets, suggesting that trochees may indeed have a processing advantage even in silent reading, irrespective of the surrounding metrical context. Finally, we note that the lexical frequency of the trochaic and iambic words was matched, thus, this pattern of results cannot be attributed to differences in frequencies.

An additional – not necessarily mutually exclusive – explanation of the present findings is that in silent reading three metrical primes were not entirely sufficient to endorse strong perception of metrical regularities and force participants to generate expectancies for the metrical pattern of the upcoming fourth word. Magne et al. (2010) who previously attempted an experiment similar to ours yet with a very low number of participants (*n* = 8), indicated that four-word contexts might suffice. They reported that the metrically incongruent fifth word enhanced negativity relative to metrically congruent one. We agree that this may be the case and therefore, as described above, interpret the effects not in terms of the perception of metrical incongruence/violation of metrical expectations but rather in the light of facilitatory role of the preceding context. Further experiments may address the issue of the length of the context sufficient to endorse perception of metrical regularities in visual language processing.

A final perspective on the attenuated negativity to incongruent items in the late time window comes from the standpoint of recent research on phonological phase boundaries. Readers, as well as listeners, seem to maintain phonological representations for about 2 s following which there is a decay of phonological information in short-term memory (Baddeley et al., 1975). In an ERP study, Roll et al. (2012) showed that when reading sentences displayed word-by-word, readers tend to insert phonological phrase boundaries at about 2.7 s which is reflected in the positivity between 500 ms and 700 ms evoked by words presented at this time point. Since items were presented every 1400 ms in the present study, readers could have processed words in units of two inserting a phonological boundary at about 2.8 ms. This means, that target words, occurring at the fourth position in a sequence, could have been perceived as phonological unit with the preceding contextual word. The phonological boundary position of the target words may have put them in focus especially in case of metrically incongruent words thereby attenuating negativity in the 400–600 ms window. One possible way of examining this explanation would be to compare ERPs evoked by the target and the preceding contextual word to fully explore to what extent phonological information span may explain the present data. However, contextual primes and targets were not adequately counterbalanced for such a comparison in the current experiment – this comparison would require that primes and targets were counterbalanced across participants such that primes and targets appeared equally often in both positions.

We have suggested a number of reasons for the attenuated negativity to iambs in incongruent relative to congruent contexts. Currently, we are unable to adjudicate between the different explanations especially since many of these explanations are not mutually exclusive. Further research is, therefore, required to provide more conclusive reasons for the pattern reported here.

Regardless of the direction of attenuated negativity, we note that the systematic differences between conditions, especially with regard to iambs in congruent and incongruent contexts allows us to draw strong conclusions regarding the activation of prosodic information during silent reading. Especially taken together with our previous study (Kriukova and Mani, 2016), the current study strongly suggests that phonological information related to the metrical structure of words is co-activated during silent reading. In a task where participants were not required in any way to attend to this prosodic content of words, we found, nevertheless a strong influence of metrical information on lexical processing. With regard to our specific aims in the current study, we found that metrical content impacts processing of words on a lexico-semantic level, although we were unable to draw stronger conclusions regarding the separability of metrical and lexico-semantic processes, given the similarity in the pattern of results obtained from the early N325 and N400 components. We suggest that it is likely that there is distributed access to information from metrical and lexico-semantic levels leading to lexical activation, and that metrical information, like other more critical sources of information about the identity of the word is processed in parallel during lexical access.

## Author Contributions

OK and NM conceptualized and designed the experiment. OK implemented the experiment, collected and analyzed the data. Both authors participated in drafting the manuscript. The manuscript was approved by both authors.

## Conflict of Interest Statement

The authors declare that the research was conducted in the absence of any commercial or financial relationships that could be construed as a potential conflict of interest.
